# Impact of Social Interaction on Customer Engagement in China’s Social Commerce—A Moderated Chain Mediation Model

**DOI:** 10.3390/bs13070541

**Published:** 2023-06-28

**Authors:** Xuexin Li, Ligang Tian, Shulin Wang

**Affiliations:** 1 Business School, Faculty of Economics, Liaoning University, Shenyang 110136, China; lixuexin@lnu.edu.cn (X.L.); tianligang@yku.edu.cn (L.T.); 2 School of Economics and Management, Yingkou Institute of Technology, Yingkou 115014, China; 3 Sunwah International Business School, Faculty of Economics, Liaoning University, Shenyang 110136, China

**Keywords:** social commerce, social interaction, social presence, customer trust, customer engagement, self-construal, China

## Abstract

With the emergence of social commerce, customer engagement is increasingly considered as an important influencing factor for enterprises to maintain a competitive advantage. Despite the extensive literature examining the determinants of customer engagement in social commerce from the perspectives of platform functions and technical dimensions, discussions on social interaction remain rare. Based on a sample dataset of 460 valid questionnaires collected via an online survey within China, using the structural equation model, this study attempts to investigate the effect of social interaction on customer engagement. Specifically, it divides social interaction into two dimensions, namely information-oriented and relation-oriented interactions. It is found that both informational and relational interactions are essential for driving customer engagement. Social presence and customer trust sequentially mediate the effect of social interaction to enhance customer engagement. In other words, social interaction enhances the sense of social presence, which in turn heightens customer trust, ultimately spurring a greater customer engagement. Self-construal moderates the relationship between social interaction and customer engagement. For interdependent customers, the effect of social interaction on customer trust is particularly significant. This study provides novel insights into how and when social interaction shapes customer engagement, highlighting the mechanisms and boundary conditions involved in this relationship within a social commerce context, which can also offer practical guidance for platforms and merchants seeking to facilitate greater engagement among customers.

## 1. Introduction

With the rapid development of e-commerce technology, online shopping has increasingly become the main method for people to purchase goods. Consumer demand shows a trend toward entertainment and interactivity. People’s shopping activities are increasingly integrated with their social activities [1]. Accordingly, various e-commerce platforms have increasingly improved their social functions by enhancing their social and community features, and diverse social media sites have also attempted to innovate more business or commercial functions by incorporating more e-commerce and online shopping features. These have spurred the emergence of social commerce [1]. Unlike traditional e-commerce with limited, lagging and imbalanced communication, in social commerce, participants can communicate instantly, inquire about product details, and check pricing, shipping and other relevant information in real-time, thereby improving transactional efficiency and reducing information asymmetry [2]. In addition, the improved interactivity also enhances the experiential pleasure for shoppers throughout their purchasing process. According to the survey, approximately 63% of respondents aged 18 to 34 regularly view live-streaming [1]. Around 79% of marketers believe that social commerce facilitates businesses to interact with consumers in a more genuine manner [2]. It is expected that by 2027, the value of global social commerce will reach USD 184.27 billion [3].

Social commerce seamlessly integrates social elements and commercial activities within a single domain, achieving business functions through social platforms [4]. Using internet technologies, social commerce enables consumers to interact with businesses more rapidly, easily and promptly throughout each phase of the purchasing journey, thereby diminishing consumers’ perceptions of uncertainty about companies, brands and products [5], eliminating psychological distance, and fully satisfying consumers’ social needs. Many researchers believe that a key prerequisite for successful social commerce is to carefully design an engaging customer journey, and it is of great importance to incorporate sufficient social interaction into each phase of the social commerce experience [4,5,6]. Studies indicate that social interaction in social commerce can not only promote customers’ understanding of product details and the feeling of realistic shopping [7], but also facilitate communication with each other, reduce perceptions of risk and generate pleasure [8,9,10], thereby further influencing customer psychology and behavior. As social interaction plays an important role in shaping consumer mind and conduct, understanding the impact of social interaction in social commerce is critical to fully tapping marketing potentials.

Customer engagement is an important concept to measure the change of a customer’s psychological state in social commerce [4,5,6]. Engaged customers are important resources for enterprises to maintain a competitive advantage. Accordingly, the research on the driving factors of customer engagement in the social commerce context has attracted widespread attention from academia. Previous studies have shown that the drivers of customer engagement in social commerce stem primarily from two aspects: technology and social factors. For example, Wongkitrungrueng et al. [5] pointed out that many individual sellers use live-streaming as direct selling tools on social commerce platforms, effectively promoting customer engagement. Wang et al. [8] believed that the technical configurations of social platforms can influence the customer experience during live-streaming viewing, hence impacting customer engagement. He et al. [11] reported that the attributes of social media brand profile pages have a positive impact on customer engagement behavior. Li et al. [12] demonstrated that network service scenarios positively influence customer engagement. Pongpaew et al. [13] examined the Facebook live-streaming scenario, and concluded that optimizing social features is conducive to customer brand engagement. Social factors also play an important role in customer engagement. Lin et al. [4] found that a happier anchor will make the audience happier and trigger a stronger audience engagement behavior. Zhang et al. [14] pointed out that the quality of user-generated content positively affects customer brand engagement. Fan et al. [15] also agreed that inter-customer social support exerts a favorable influence on customer engagement behavior. Xue et al. [16] claimed that on-site interaction stimulates perceived usefulness, while mitigating perceived risk and psychological distance, thereby promoting customer engagement in social commerce. Samala et al. [17] pointed out that customer participation has a positive effect on customer brand engagement. Samarah et al. [18] suggested that customers’ brand interactivity and involvement positively impact their own brand engagement on social media. Fei et al. [19] believed that the interaction between anchors and consumers promotes customers’ consumption behavior. Guo et al. [20] claimed that because the anchor plays the role of both product salesperson and opinion leader, his or her personal characteristics will affect customer engagement.

While previous research has explored drivers of customer engagement in social commerce, most studies have focused narrowly on platform features and technical elements. Although some research has examined the link between interaction and customer engagement, only a few studies have primarily emphasized human–computer interaction [5,8,12], like how platform tools and service models impact customer engagement, as well as anchor attributes, such as upbeat personalities or opinion leadership, to drive customer engagement [4,19,20]. These studies tend to treat customers as passive recipients of interactions, ignoring their purpose in participating on social commerce platforms. The motivations driving customers to engage on these platforms extend far beyond product information and include fulfilling their social and relational needs, thereby generating a high-quality customer experience [8,9,10].

Therefore, it is insufficient to explore the impact of social interaction on customer engagement by solely focusing on the source of interactive content (e.g., platforms or influencers). Research must also consider the purposes motivating customers to participate in social commerce. Social interaction encompasses both informational and relational content [10,21]. While interactions may convey product details or recommendations, they also satisfy customers’ needs for connection and shared purpose. Based on this, this article divides social interaction into information-oriented and relationship-oriented interactions [9,10], and deeply explores the impact of social interaction on customer engagement in the context of social commerce from these two dimensions.

Meanwhile, social presence and customer trust play indispensable roles in the relationship between social interaction and customer engagement [13,18,22,23,24]. The perception of social presence depends on interactive factors [23,25] and significantly impacts customers’ shopping decisions online [26,27]. Research shows that social presence and customer trust play a key role in cultivating customer engagement. For example, Samarah et al. [18] found that customer trust mediates the relationship between brand interaction and customer engagement. Pongpaew et al. [13] demonstrated that customer engagement, perceived social presence, and brand trust are closely linked in social commerce, shaping customers’ attitudinal and behavioral loyalty. Therefore, social presence and customer trust are critical to understanding how customer engagement forms. The pursuit of authenticity and presence is an important reason why customers involve in social commerce. Social commerce’s interactive features can enable customers to experience interactive social feelings during viewing live-streaming, thereby creating a sense of social presence. This can improve interpersonal relationships, form customer trust, and increase customer engagement [5,24]. Therefore, this study examines the chain mediating roles of social presence and customer trust in the relationship between social interaction and customer engagement.

In addition, the impact of social interaction on customer engagement may vary depending on individuals’ way of thinking [5]. Self-construal explains differences in individuals’ thinking styles. Compared to those with independent self-construal, individuals with interdependent self-construal show more group dependence, are more easily influenced by social interaction, and are more prone to customer engagement [28]. Therefore, this study also investigates the moderating role of self-construal in the relationship between social interaction and customer engagement.

This study aims to provide some novel insights into how and when social interaction shapes customer engagement within a social commerce context, which can also offer practical guidance for platforms and merchants seeking to facilitate greater engagement among customers. Our research contributes to the extant literature in three aspects. First, it extends the customer engagement research scope into the social commerce context. Contrasted with conventional commerce settings, the antecedents of customer engagement may diverge within a social commerce context. It enriches the studies of customer engagement by investigating the antecedents of customer engagement in the social commerce context. Second, this paper examines the effect of social interaction on customer engagement. Despite the existing literature examining the determinants of customer engagement in social commerce from technological and social perspectives, most studies focus on platform features and technical elements. Discussions from the perspective of social interaction are insufficient. This paper examines the impact of social interaction (including information-oriented and relationship-oriented interactions) on customer engagement, thereby augmenting the understanding of customer engagement. Third, this paper attempts to explore the underlying mechanism and the boundary condition of social interaction on customer engagement. Specifically, it examines the chain mediating effect of social presence and customer trust, as well as the moderating influence of self-construal, on the relationship between social interaction and customer engagement. It enhances our insight into the mechanism whereby social interaction exerts its effect.

## 2. Theoretical Analysis and Hypothesis Development

### 2.1. Social Interaction and Customer Engagement

Social interaction can be broadly defined as any behavior wherein individuals participate, and it can sway other consumers’ assessments or decisions regarding a product or service [10]. In particular, social interaction refers to communication and interchanges among individuals [21]. People may influence each other’s decisions through interactions [10,21]. According to the purpose of interactions, social interaction can be further divided into two categories, namely information-oriented and relationship-oriented social interactions [9]. The stimulus-organism-response (SOR) model, adapted from psychology, proposes that environmental stimuli indirectly shape human behavior by influencing internal psychological states. According to this theory, external stimuli (S) activate certain organism (O) variables—emotions, cognitions and motivations—which then drive behavioral responses (R) [29,30]. Applied to customer experience, social interaction acts as a key stimulus influencing customer psychology and behavior, thereby exerting influence on the customer’s shopping experience and customer engagement [31,32]. Customer engagement refers to the intensity of customer participation and connection to the organization’s offerings or activities, reflecting the customer’s recognition of the company’s products and services [4,5]. In a social commerce context, customer engagement can be understood as the extent to which customers devote themselves to the social platform according to their own preferences and interests. It is an important influencing factor for enterprises to maintain competitive advantage.

In social commerce, social interaction is an important way for customers to participate in marketing activities [8,9,10]. Customers communicate through chatting, commenting, bullet screens, expressions, voice chat and other forms. In this way, customers in the same live-streaming room form a group. On the one hand, customers share product-related information and their shopping experience through interaction, namely information-oriented social interaction. It facilitates customers in formulating initial evaluations of merchants, which serve as a precondition for their shopping choices. The information-oriented social interaction can greatly eliminate customers’ uncertainty perception of companies, brands and products [5], and heighten the precision of shopping recommendation provided to customers. On the other hand, as customers gain a profound comprehension of product information, it will spur their interaction with anchors and other customers, specifically in the form of relationship-oriented social interaction. Harmonious relationship-oriented interaction results in pleasure, intimacy and trust for customers in social commerce [4,5,6,7], which helps customers generate positive shopping behaviors, such as brand recommendation, active participation in live-streaming room activities, writing feedback reviews, etc. [9,10].

Based on the above analysis, we believe that in the social commerce context, information-oriented interaction facilitates customer understanding of the product information, thus reducing information asymmetry and constituting precise expectations of product and service quality. Relationship-oriented interaction can catalyze the accrual of emotional experiences and sentiments and create a more harmonious shopping environment to promote customer engagement. Therefore, we propose the following hypotheses:

**Hypothesis** **1a** **(H1a):** *Information-oriented interaction positively influences customer engagement.*

**Hypothesis** **1b** **(H1b):** *Relationship-oriented interaction positively influences customer engagement.*

### 2.2. The Mediating Role of Social Presence

Social presence refers to the extent to which a medium allows a person to be perceived as present while interacting [25]. Some scholars point out that social presence exerts substantial impact in raising customers’ sense of security and affirmative consumption attitudes within the virtual shopping experience, thereby constituting a core determinant motivating customers to choose to shop through online platforms [23,26,27]. Zhao et al. [24] showed that online interaction on business-to-customer websites boosts customers’ social presence. Gao et al. [33] ascertained that bullet screen interaction among viewers in live-streaming positively affects social presence. In contrast with conventional e-commerce, which must depend exclusively upon words and pictures to convey information, in social commerce, businesses display product information through diverse forms such as sound, video, text and pictures, allowing customers to experience social presence with others during the communication process. Meanwhile, upon watching live-streaming, customers can communicate with businesses or other customers. Relationship-oriented interaction enhances emotional exchange between businesses and customers, which further reduces customers’ perception of uncertainty, increases customer involvement, and proffers an immersive experience to customers, thus producing a shopping experience similar to that within a realistic setting. Therefore, we propose the following hypotheses:

**Hypothesis** **2a** **(H2a):** *Information-oriented interaction positively influences social presence.*

**Hypothesis** **2b** **(H2b):** *Relationship-oriented interaction positively influences social presence.*

Social presence empowers customers to undergo a shopping experience almost exactly like that achieved in a realistic environment. This effectually overcomes the sense of distance intrinsic to online shopping, creating a psychological familiarity and cordiality toward consumers, and generates more enthusiasm for consumers to involve themselves in interaction. In social commerce, consumers experiencing a strong feeling of social presence concentrate more intensely and process information more efficiently [33]. This results in a more satisfactory shopping experience to promote customer engagement. To sum up, information interaction fosters customers’ understanding of the product details, and relation-oriented interaction promotes the emotional exchanges in social commerce. Together, they provide an immersive real experience, thereby enhancing social presence. A strong social presence increases customers’ attention to businesses, and drives deep customer engagement. Therefore, we propose the following hypotheses:

**Hypothesis** **3a** **(H3a):** *Social presence mediates the relationship between information-oriented interaction and customer engagement.*

**Hypothesis** **3b** **(H3b):** *Social presence mediates the relationship between relation-oriented interaction and customer engagement.*

### 2.3. The Mediating Role of Customer Trust

Customer trust manifests customers’ affirmation of the technology and services provided by the social commerce platform, as well as their recognition of other customers [34,35,36]. Customer trust constitutes a critical concern within the online shopping context and particularly plays an important role in social commerce [18,22]. In accordance with the SOR model, customer trust is formed in the process of communication between buyers and sellers, and is affected by many factors. Extant studies have shown that online interaction [24], bullet screen interactivity [33], scenario promotion [33], and the interactive characteristics of e-commerce anchors [37] all have a positive impact on customer trust. In social commerce, customers share product information and provide real-time feedback on product usage by engaging in information-oriented interaction. This validates the marketing messages conveyed by businesses, and thus increases the customer trust. Similarly, by means of relation-oriented interaction, customers enhance friendship and emotional connections, and customers’ trust in businesses increases. Therefore, we propose the following hypotheses:

**Hypothesis** **4a** **(H4a):** *Information-oriented interaction positively influences customer trust.*

**Hypothesis** **4b** **(H4b):** *Relationship-oriented interaction positively influences customer trust.*

In social commerce, customer trust is an important variable to judge whether the relationship between customers and businesses is harmonious. Social interaction helps to enhance customer trust. In turn, customer trust makes customers eager to continue using what companies offer [38] and create longer-lasting relationships between businesses and customers. Previous research has proven that customer engagement is influenced by customer trust [5,12,18]. In social commerce, customer trust improves the relationship between customers and businesses, which is conducive to enhancing customer engagement.

In summary, information-oriented interaction enables customers to have an in-depth understanding of businesses’ credibility, commitments and their recommended products, eliminating information asymmetry between buyers and sellers. Relation-oriented interaction enhances the emotional connection between customers and businesses. Both types of social interactions improve the customer experience as well as increase customers’ trust in businesses, and thus promotes customer engagement. Therefore, we propose the following hypotheses:

**Hypothesis** **5a** **(H5a):** *Customer trust mediates the relationship between information-oriented interaction and customer engagement.*

**Hypothesis** **5b** **(H5b):** *Customer trust mediates the relationship between relation-oriented interaction and customer engagement.*

### 2.4. A Chain Mediating Role of Social Presence and Customer Trust

Customers in the same live-streaming room constitute an interconnected system of information exchange. Their behaviors will affect other customers [33]. Social interaction can shorten the distance between customers, reduce information asymmetry between buyers and sellers, result in an immersive sense of reality to each participant, enhance customers’ sense of social presence, and increase customers’ trust in businesses [24,33,39]. Previous studies have shown that social presence plays a mediating role in the relationship between online interaction and customer trust [24]. Particularly, on the one hand, consumer interactions with websites, sellers, and other consumers can enhance customers’ sense of social presence. On the other hand, the sense of social presence with fellow live-streaming viewers boosts customers’ trust [33]. When there is a strong social interaction in social commerce, customers tend to have a strong sense of social presence, which can reduce customers’ perceived risk and psychological distance, thereby enhancing customers’ trust in businesses. Therefore, we propose the following hypothesis:

**Hypothesis** **6** **(H6):** *Social presence positively influences customer trust.*

Combining H3a, H3b, H5a, H5b and H6, we further propose H7a and H7b.

**Hypothesis** **7a** **(H7a):** *Social presence and customer trust play a chain mediating role between information-oriented interaction and customer engagement.*

**Hypothesis** **7b** **(H7b):** *Social presence and customer trust play a chain mediating role between relation-oriented interaction and customer engagement.*

### 2.5. Moderating Role of Self-Construal

According to the SOR model, individuals respond to the same stimuli differently, which is closely related to individuals’ own characteristics. Self-construal is precisely the construct used to explain the differences in individuals’ own characteristics. Self-construal refers to individuals’ cognition and views on the relationship between self and others. It consists of two types: independent self-construal and interdependent self-construal [40]. Individuals with different self-construal types reveal substantial discrepancies within their cognitive modes. In particular, individuals with interdependent self-construal prefer to attach themselves to the collective [28], thus generating a sense of security, and tend to obey the common choice of the collective. However, individuals with independent self-construal tend to focus on themselves and rely on their own feelings to make judgements and decisions [40,41]. Therefore, customers with different self-construal types will have different psychological reactions to social interaction.

Individuals with interdependent self-construal can perceive the commonality between themselves and others when making social comparisons, and tend to believe that their goals are similar to those of others [41]. They tend to be heavily swayed in their own decision-making by the information and opinions offered by others [42]. Previous studies have confirmed that customers with interdependent self-construal have a higher willingness to engage online when there are more people involved in the interaction or community [43] and are more inclined to rely on others to make decisions in order to avoid risks [42]. In contrast, those with independent self-construal view themselves as autonomous and self-sufficient. For independent customers, the number of people involved has little impact on willingness to engage or the perceived costs of doing so. They tend to rely on personal priorities to evaluate options rather than the preferences or behaviors of others [43].

Compared with individuals with independent self-construal, those with interdependent self-construal pay more attention to environmental factors. They readily adapt their behavior to match environmental changes. They aim to maintain choices consistent with the people around them, tend to follow others’ recommendations, and ultimately engage in similar buying patterns. In the context of social commerce, customers with interdependent self-construal are more easily influenced by social interactions, resulting in a strong sense of social presence. Meanwhile, they are more willing to accept the suggestions of businesses and other customers, thereby boosting customer trust. Therefore, we propose the following:

**Hypothesis** **8a** **(H8a):** *Self-construal moderates the relationships between information-oriented interaction and social presence. Specifically, for customers with interdependent self-construal, the influence of information-oriented interaction on social presence is more significant.*

**Hypothesis** **8b** **(H8b):** *Self-construal moderates the relationships between relation-oriented interaction and social presence. Specifically, for customers with interdependent self-construal, the influence of relation-oriented interaction on social presence is more significant.*

**Hypothesis** **8c** **(H8c):** *Self-construal moderates the relationships between information-oriented interaction and customer trust. Specifically, for customers with interdependent self-construal, the influence of information-oriented interaction on customer trust is more significant.*

**Hypothesis** **8d** **(H8d):** *Self-construal moderates the relationships between relation-oriented interaction and customer trust. Specifically, for customers with interdependent self-construal, the influence of relation-oriented interaction on customer trust is more significant.*

Combining H7 and H8, we further construct a moderated chain mediation model. In particular, the mediating effect of social presence and customer trust on the relationship between social interactions and customer engagement is influenced by the type of self-construal. Therefore, we propose the following:

**Hypothesis** **9a** **(H9a):** *Self-construal moderates the chain mediating effect of social presence and customer trust on the relationship between information-oriented interaction and customer engagement. Specifically, for customers with interdependent self-construal, the chain mediating effect is more significant.*

**Hypothesis** **9b** **(H9b):** *Self-construal moderates the chain mediating effect of social presence and customer trust on the relationship between relation-oriented interaction and customer engagement. Specifically, for customers with interdependent self-construal, the chain mediating effect is more significant.*

**Figure 1:**
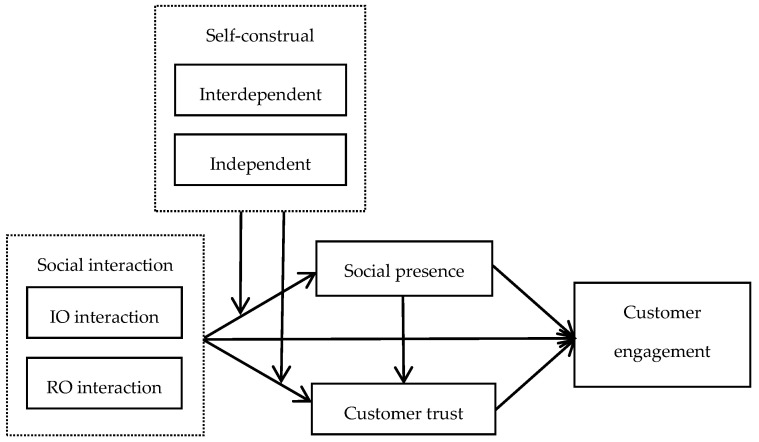
Theoretical model of the study.

Based on the above hypotheses, we construct our theoretical model as shown in Figure 1. It illustrates the relationships among information-oriented interaction (IO), relation-oriented interaction (RO), social presence, customer trust, customer engagement, and self-construal.

## 3. Methods

### 3.1. Data Collection

Before developing the survey, we consulted 10 subject experts to evaluate the rigor of our design and data collection methodology. We also interviewed 34 social commerce users aged 16 to 62 via both online and face-to-face discussions. These interviews aimed to gain deep insight into how customers engage and interact within social commerce communities, their psychological traits and mindsets that shape their participation, as well as their feedback to refine survey questions for clarity and comprehension. Through these consultations and interviews, we identified that the more experienced social commerce users tend to range from 26 to 35 years of age.

The survey was conducted via the online website Wenjuanxing (https://www.wjx.cn, accessed on 10 September 2022). Wenjuanxing is China’s leading online survey platform. Two primary methods are used for sample collection: one is collecting data directly from the Wenjuanxing survey platform, and the other is circulating the questionnaire link on the Chinese social platform, Wechat, and inviting Chinese customers to participate. Individuals cannot respond to the survey more than once, and respondents with the same IP address can only complete the questionnaire once.

The questionnaire comprises three sections (see Supplementary Materials File S1 for details). The first section contains two questions. One is the filter question. After reading the information describing social commerce platforms, participants were asked whether they have experience participating in social commerce platforms (including registration/browsing/posting/commenting/participating in interactions/shopping). Those without experience on these platforms are not eligible respondents. The other question directs respondents to specify the type of social commerce platforms they are most familiar with, and then respond to the rating scales based particularly on experiences and perspectives related to that platform. The second section of the questionnaire contains five 7-point rating scales, and in the third section, respondents’ demographic data are collected. Data collection spanned from 10 September to 20 December 2022. In total, 510 questionnaires were distributed. Following screening of responses for validity, 460 complete and valid responses were collected, indicating an effective response rate of 90.2%. The demographic characteristics of the sample are described in Table 1.

### 3.2. Measurement of Variables

Social interaction: Social interaction consists of two dimensions, namely information-oriented interaction and relationship-oriented interaction. Referring to Zhou [9] and Liu et al. [10], the scale is designed with a total of 7 items, of which the first 4 items are used to measure information-oriented interaction (e.g., “Social commerce platforms make it effortless for me to access useful product information.”). The last 3 items are used to measure relationship-oriented interaction (e.g., “I frequently connect through open dialogue with others on the social commerce platforms, bonding over shared interests and building relationships.”).

Customer engagement: Following the scale developed by Wongkitrungrueng et al. [5], we design a 3-item scale to measure the construct of customer engagement (e.g., “When I need to shop, social commerce platforms come to mind for me.”).

Social presence: Social presence scale was adopted from Lee et al. [23], with three items (e.g., “Social commerce gives me a sense of being immersed and present.”).

Customer trust: The scale of customer trust was adopted from Chen et al. [22], containing three items (e.g., “I feel that the offerings from merchants on social commerce platforms have a guaranteed quality.”).

Self-construal: The measurement of self-construal refers to the scale developed by Singelis [40] and Tu et al. [44], with a total of 3 items (e.g., “I am easily influenced by other people’s ideas.”). A higher score indicates that the respondent shows a higher level of interdependent self-construal, while a lower score represents an independent self-construal. All scales adopt a Likert 7-point rating, with 1–7 representing the degree of agreement from low to high.

## 4. Results

### 4.1. Testing of Reliability and Validity

This study used AMOS 23.0 and SPSS 23.0 to analyze the sample data. Firstly, the results of the reliability test are reported in Table 2. The Cronbach’s alpha reliability coefficients for all variables ranged from 0.829 to 0.888, while the composite reliability (CR) coefficients ranged from 0.829 to 0.889, exceeding the reference value of 0.7. This indicates that the scales had acceptable reliability [45]. Second, this study employed confirmatory factor analysis (CFA) to ascertain the goodness of fit of the overall model. The CFA results demonstrated a relatively good fit between model and data (χ^2^/df = 1.253, lower than the reference value of 3; NFI = 0.967, RFI = 0.959, IFI = 0.993, TLI = 0.992, CFI = 0.993, exceeding the acceptable value of 0.9; RMSEA = 0.023, lower than the reference value of 0.05) [46]. Third, as shown in Table 2, the average variance extracted (AVE) exceeded 0.5 for all variables [45], and the standardized factor loadings were greater than 0.7 for all items, well above the acceptable threshold of 0.5 [47]. This indicates that the scales exhibited adequate convergent validity and were acceptable. In addition, the results of discriminant validity testing are shown in Table 3. The square roots of AVE ranged from 0.786 to 0.816 for the variables, while their inter-correlations ranged from 0.213 to 0.713. The square roots of the AVE were greater than the inter-construct correlations [48], thereby exhibiting discriminant validity. Finally, the scales used in the research were adapted from rigorously developed measures established in prior work. We carefully modify the wording of these scales, taking expert suggestions, characteristics of the subjects, research objectives, and environmental factors into consideration. Therefore, the scales demonstrated good content validity.

### 4.2. Common Method Biases

This study uses two approaches to conduct a common method bias test, namely Harman single factor test and correlation coefficient between latent variables. First, an exploratory factor analysis was performed on all questionnaire items. The first factor explains 25.83% of the total variation, lower than the critical value of 50%, indicating that there was no significant common method bias in the research data. Second, the correlation coefficients between latent variables ranged from 0.213 to 0.713, all less than 0.9 (see Table 3 for details). Combining the results of the two tests, it can be known that the common method bias is not significant.

### 4.3. Hypothesis Testing

This study establishes a structural equation modelling (SEM) to test the direct effects between variables. The results are presented in Table 4. Both the coefficients of information-oriented (IO) and relationship-oriented (RO) interactions on customer engagement are significant (β = 0.397, *p* < 0.001; β = 0.370, *p* < 0.001), indicating that information-oriented and relationship-oriented interactions positively impact customer engagement. Therefore, Hypotheses H1a and H1b are supported. Meanwhile, both information-oriented and relationship-oriented interactions have a significant positive impact on social presence (β = 0.425, *p* < 0.001; β = 0.350, *p* < 0.001), and thus, Hypotheses H2a and H2b are supported. In addition, the coefficients of information-oriented and relationship-oriented interactions on customer trust are significant (β = 0.345, *p* < 0.001; β = 0.399, *p* < 0.001), indicating that both types of social interaction positively influence customer trust. Therefore, Hypotheses H4a and H4b are supported. Finally, social presence positively impacts customer trust (β = 0.561, *p* < 0.001). Therefore, hypothesis H6 is supported.

Next, we tested the mediating role of social presence and costumer trust using structural equation modeling (SEM) with maximum likelihood estimation. More precisely, we conducted percentile bootstrapping as well as bias-corrected percentile bootstrapping with 5000 resamples to construct 95% confidence intervals for the indirect effects. We examined the confidence interval bounds to determine whether the indirect effects were statistically significant based on the criteria proposed by Preacher and Hayes [49].

**Figure 2:**
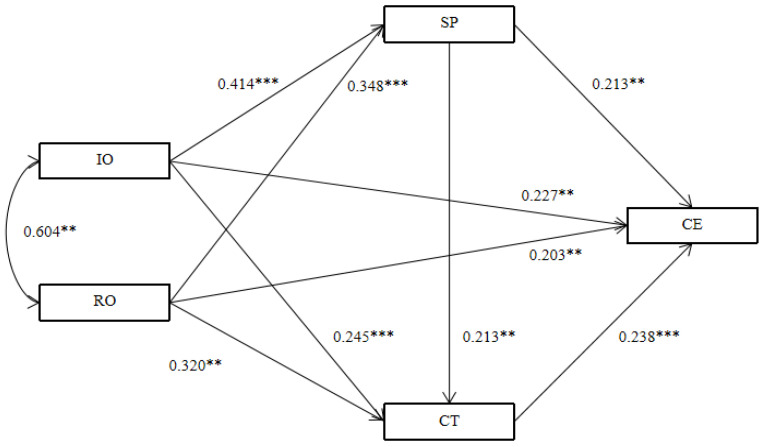
Mediation effect model of information-oriented interaction, relationship-oriented interaction, social presence, customer trust, customer engagement. N = 460, ** *p* < 0.01, *** *p* < 0.001.

The CFA results demonstrated a relatively good fit between the mediating effect model and data (χ^2^/df = 1.331, NFI = 0.973, RFI = 0.966, IFI = 0.993, TLI = 0.991, CFI = 0.993, RMSEA = 0.027) [46]. As illustrated in Figure 2, information-oriented interaction positively influences social presence (β = 0.414, *p* < 0.001); relationship-oriented interaction positively influences social presence (β = 0.348, *p* < 0.001); and social presence positively influences customer engagement (β = 0.213, *p* < 0.01). Information-oriented interaction positively affects customer trust (β = 0.245, *p* < 0.001); relationship-oriented interaction positively affects customer trust (β = 0.320, *p* < 0.01); customer trust positively affects customer engagement (β = 0.238, *p* < 0.001); and social presence positively affects customer trust (β = 0.213, *p* < 0.01). The direct effect results of information-oriented interaction on customer engagement (β = 0.227, *p* < 0.01) as well as the relationship-oriented interaction on customer engagement (β = 0.203, *p* < 0.01) are both statistically significant.

The bootstrap results are presented in Table 5. Mediating effects of social presence are statistically significant for both the relationship between information-oriented interaction and customer engagement (β = 0.090, *p* < 0.001), as well as between relationship-oriented interaction and customer engagement (β = 0.074, *p* < 0.001). Thus, Hypotheses 3a and 3b are supported. In addition, the mediating effects for customer trust are statistically significant for both the relationships between information-oriented interaction and customer engagement (β = 0.059, *p* < 0.001) as well as between relationship-oriented interaction and customer engagement (β = 0.076, *p* < 0.001), which are all statistically significant. Therefore, Hypotheses 5a and 5b are supported. Finally, the chain mediating effects for social presence and customer trust between information-oriented interaction and customer engagement (β = 0.021, *p* < 0.01), and between relationship-oriented interaction and customer engagement (β = 0.018, *p* < 0.001) are statistically significant. Therefore, Hypotheses 7a and 7b are supported.

We used model 1 of the PROCESS macro for SPSS to test the moderating role of self-construal. As recommended by Cohen et al. [50] and Meng et al. [51], we first centered the variables to mitigate multicollinearity. Subsequently, we calculated the mean and standard deviation of self-construal based on the centered data. Scores below one standard deviation of the mean were designated as the independent self-construal group, and the scores above one standard deviation of the mean were designated as the interdependent self-construal group. Using these self-construal group categorizations, we then tested for moderated effects. The independent and interdependent groups enabled us to examine how the relationships between variables might differ based on self-construal type.

**Figure 3:**
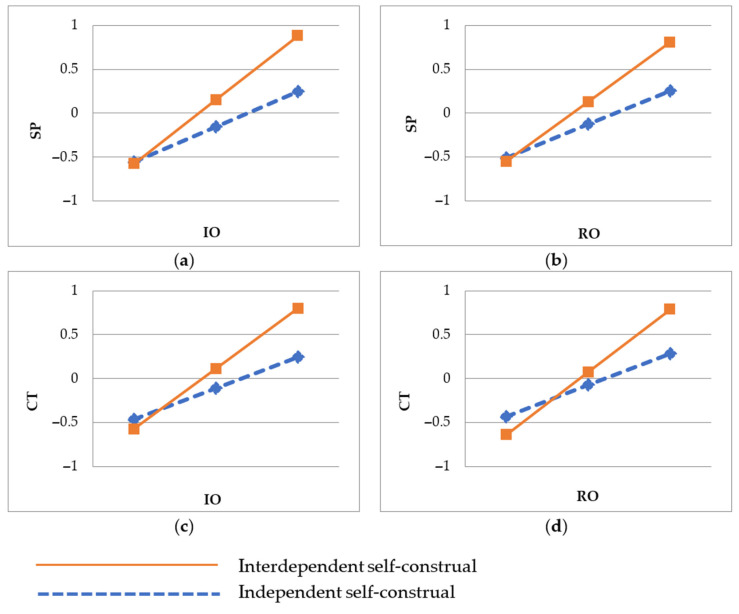
The moderating effects of self-construal on (**a**) the relationship between IO and SP; (**b**) the relationship between RO and SP; (**c**) the relationship between IO and CT; (**d**) the relationship between RO and CT. N = 460.

The results are shown in Table 6. It indicates that self-construal moderated the relationships between information-oriented interaction, relationship-oriented interaction, and social presence; self-construal also moderated the relationships between information-oriented interaction, relationship-oriented interaction, and customer trust. Specifically, for customers with interdependent self-construal, the influence of social interaction (IO: β = 0.834; RO: β = 0.775) on social presence is more significant compared with those with independent self-construal (IO: β = 0.296; RO: β = 0.286), wherein the 95%CI do not contain zero. Therefore, Hypotheses 8a and 8b are supported. Meanwhile, for interdependent self-construal, the influence of social interaction (IO: β = 0.796; RO: β = 0.826) on customer trust is more significant than that of independent self-construal (IO: β = 0.246; RO: β = 0.248), wherein the 95%CI do not contain zero. Hypotheses 8c and 8d are thus supported. Figure 3 illustrates the slopes of these four moderating effects.

To further examine the moderated mediation with a chain of effect, we used model 84 of the PROCESS macro for SPSS to conduct the analyses. As shown in Table 7, the self-construal type moderated the relationships of information-oriented interaction and relationship-oriented interaction with social presence. The self-construal type also moderated the relationships of information-oriented interaction and relationship-oriented interaction with customer trust. Furthermore, self-construal type moderated the chain mediating effects of social presence and customer trust on the relationships of information-oriented interaction and relationship-oriented interaction with customer engagement. Specifically, for individuals with interdependent self-construal, social presence had stronger mediating effects on the influence of information-oriented interaction and relationship-oriented interaction on customer engagement (β = 0.221; β = 0.222) compared to individuals with independent self-construal (β = 0.078; β = 0.082), wherein all 95%CI do not contain zero. This provided further support for Hypotheses 8a and 8b. Similarly, for interdependent self-construal, customer trust had stronger mediating effects on the influence of information-oriented interaction and relationship-oriented interaction on customer engagement (β = 0.176, β = 0.183) compared with those of independent self-construal (β = 0.052, β = 0.051), further supporting Hypotheses 8c and 8d. Finally, the chain mediating effects of social presence and customer trust were significant and more robust for individuals with interdependent (β = 0.056, β = 0.050) compared to independent self-construal (β = 0.020, β = 0.019), with all 95%CI not containing zero, thereby supporting Hypotheses 9a and 9b. The results of hypothesis testing are summarized in Table 8.

## 5. Discussion

First, this study finds that social interactions spur customer engagement in social commerce, which is consistent with previous works [9,10,16]. Specially, the finding that relationship-oriented social interaction impacts customer engagement confirms previous research by Samarah [18] and Kang et al. [52]. Moreover, this study also finds that information-oriented interaction significantly drives customer engagement within social commerce environments, thus enriching theoretical research on the customer engagement formation mechanism.

Second, this study reveals the path mechanism through which social interaction influences customer engagement in social commerce. Most prior research has recognized that environmental factors impact the emergence of customer engagement, and social presence and customer trust as antecedents of customer engagement have also been verified [5,13,18,20,24]. This research unified social interaction, social presence, and customer trust and engagement within a single model to examine their interdependent role in shaping customer experience. The results not only validate past findings that social presence and trust drive engagement, but also extend understanding by uncovering the chain mediation process through which social interaction influences customer engagement in social commerce contexts.

Third, this study also verifies the moderating role of self-construal, thereby clarifying the boundary conditions under which social interaction influences customer engagement. Prior research emphasizes the role of environmental influences on customer engagement behaviors while largely overlooking individual differences [4,5,6]. This research found that the influence of social interaction on customer experience depends profoundly on individual differences in thinking styles. While environmental factors drive certain behaviors, individuals determine how to interpret and act on them based on their own mindsets. It enriches the understanding of self-construal by identifying it as a key moderator of external influences of social interaction on customer behaviors.

## 6. Conclusions

Based on the context of social commerce, this study provides novel insights into how and when social interaction shapes customer engagement, highlighting the mechanisms and boundary conditions involved in this relationship. It finds that both informational and relational interactions are essential for driving customer engagement. Social presence and customer trust sequentially mediate the effect of social interaction to enhance engagement. Social interaction enhances the sense of social presence, which in turn heightens customer trust, ultimately spurring a greater customer engagement. Self-construal moderates the relationship between social interaction and customer engagement. Specifically, for interdependent customers, the effect of social interaction on customer trust is particularly significant.

### 6.1. Managerial Implications

Based on our research results, this paper provides the following implications from the perspectives of social commerce platforms, social commerce merchants, and relevant public sections to improve the customer experience.

For social commerce platforms: First, since social interaction promotes customer engagement, social commerce platform companies should optimize the interactive features to facilitate customer engagement. More precisely, they can use technical means to make the interface friendlier and more humane, thereby strengthening the analysis of interactive content to identify and provide information that strongly spurs customer engagement. Second, social commerce platform companies should enhance customers’ sense of social presence by fully tapping their technical potentials and stimulating a more realistic live-streaming environment for customers. Third, social commerce platform companies should strive to increase customer trust in the platform, pay more attention to customer privacy protection, establish a mechanism to verify the authenticity of information, and build a social commerce platform with strong credibility. Fourth, social commerce platform companies should accommodate variability in customer mindsets to optimize engagement across diverse audiences. In particular, to resonate with independence-oriented customers, platforms could cultivate engagement through customization, or enable customers to choose their own sequence of interactions and connections rather than a predetermined flow of activities.

For social commerce merchants: As we have mentioned, customers’ information-oriented interaction and relation-oriented interaction will affect customer trust and customer engagement, and this relationship is moderated by customers’ self-construal type. Therefore, for merchants involving in social commerce, we have several recommendations. First, merchants should create an effective live-streaming environment that boosts social interaction. Merchants need to stimulate customers’ desire to interact and encourage customers to participate in interaction through developing appropriate topics, as well as setting up rewards and contests to create a harmonious interactive atmosphere. Second, merchants should improve anchors’ communication ability. Anchors should be able to reasonably guide customers to participate in interaction and maintain the emotional connection between anchors and customers, and among customers. In addition, for different types of customers, merchants should design different interactive methods, because the customer’s thinking style determines the way they understand and process interactive information, which is ultimately reflected in the differences in interaction effects.

For relevant government agencies, social commerce is an emerging business model that differs from traditional commerce. The existing regulatory system cannot fully adapt to its unique characteristics, potentially resulting in improper regulation or regulatory failure. Therefore, government departments should be responsible to regulate social commerce in a manner beneficial for all participants. First, government agencies should establish a comprehensive legal framework tailored for social commerce. Moreover, government agencies must monitor platforms, merchants, and other social commerce companies for violations and misconduct in order to protect customers and maintain fair market order. In addition, policy-makers should design policies that specifically promote the growth of social commerce. Finally, governments should issue recommendations centered on augmenting social interaction, customer trust, and engagement.

### 6.2. Limitations and Future Directions

Although this study has obtained some valuable conclusions, there are still some limitations. First, this study is based on the social commerce scenarios in China, which may pose an issue of generalizability. Under different cultural backgrounds, the mechanism by which social interaction affects customer engagement may differ. Therefore, future research is welcome to further explore such influence mechanisms in different cultural contexts. Second, this study explores the mediating role of social presence and customer trust in the relationship between social interaction and customer engagement, and thus may ignore some other potential path factors. Future research is also advised to explore the path influences of social interaction on customer engagement from multiple dimensions. Finally, this study focuses on exploring the boundary conditions of the influence of social interaction on customer engagement from the perspective of differences in individuals’ thinking patterns. Future research can further explore the boundary conditions from other perspectives, such as customer characteristics and geographic locations.

## Figures and Tables

**Table 1:** Sample characteristics.

Demographics	Frequency	Percentage (%)
*Gender*		
Men	203	44.13
Women	257	55.87
*Age*		
Under 18	50	10.87
From 18 to 25	103	22.39
From 26 to 35	185	40.22
From 36 to 45	71	15.43
Over 45	51	11.09
*Education*		
High school/secondary school and below	100	21.74
College degree	118	25.65
Bachelor’s degree	214	46.52
Master’s degree and above	28	6.09
*Years of watching live* *-streaming*		
Less than half a year	22	4.78
Half a year to two years	181	39.35
Two years and above	257	55.87
*Platforms’ online duration*		
Less than 1 h per day	11	2.39
1–2 h per day	70	15.22
2–3 h per day	265	57.61
More than 3 h per day	114	24.78
Total	460	100

**Table 2:** Results of reliability and convergent validity.

Construct	Item	Loading	AVE	CR	Cronbach’s Alpha
Information-oriented interaction	IO1	0.840	0.666	0.889	0.888
	IO2	0.800			
	IO3	0.799			
	IO4	0.825			
Relationship-oriented interaction	RO1	0.838	0.657	0.853	0.852
	RO2	0.797			
	RO3	0.799			
Social presence	SP1	0.805	0.665	0.856	0.856
	SP2	0.822			
	SP3	0.820			
Customer trust	CT1	0.809	0.652	0.849	0.849
	CT2	0.802			
	CT3	0.812			
Customer engagement	CE1	0.835	0.665	0.856	0.855
	CE2	0.789			
	CE3	0.822			
Self-construal	SC1	0.788	0.618	0.829	0.829
	SC2	0.791			
	SC3	0.780			

Note: N = 460; IO—information-oriented interaction; RO—relationship-oriented interaction; SP—social presence; CT—customer trust; CE—customer engagement; SC—self-construal.

**Table 3:** Results of discriminant validity.

Variable	1	2	3	4	5	6
1. Information-oriented interaction	0.816					
2. Relationship-oriented interaction	0.690 ***	0.812				
3. Social presence	0.713 ***	0.698 ***	0.816			
4. Customer trust	0.656 ***	0.697 ***	0.651 ***	0.808		
5. Customer engagement	0.701 ***	0.710 ***	0.702 ***	0.709 ***	0.816	
6. Self-construal	0.213 ***	0.304 ***	0.301 ***	0.248 ***	0.260 ***	0.786

Note: N = 460; underlined values on the diagonal are the square roots of the AVE; *** *p* < 0.001.

**Table 4:** Results of structural equation model (SEM).

Path	Standardized Coefficient	t Value
IO Interaction→Customer Engagement	0.397 ***	9.116
RO Interaction→Customer Engagement	0.370 ***	8.682
IO Interaction→Social Presence	0.425 ***	9.670
RO Interaction→Social Presence	0.350 ***	8.139
IO Interaction→Customer Trust	0.345 ***	7.499
RO Interaction→Customer Trust	0.399 ***	8.863
Social Presence→Customer Trust	0.561 ***	14.360

Note: N = 460, *** *p* < 0.001.

**Table 5:** Bootstrap analysis of mediation effects.

	Point Estimate	SE	Z	Bias-Corrected 95%CI		Percentile 95%CI	
				Lower	Upper	Lower	Upper
*Indirect effects*							
IO→SP→CE	0.090 **	0.028	3.214	0.041	0.156	0.040	0.152
RO→SP→CE	0.074 **	0.021	3.524	0.036	0.120	0.034	0.117
IO→CT→CE	0.059 ***	0.021	2.810	0.026	0.109	0.023	0.105
RO→CT→CE	0.076 ***	0.022	3.455	0.039	0.125	0.037	0.121
IO→SP→CT→CE	0.021 **	0.009	2.333	0.008	0.044	0.007	0041
RO→SP→CT→CE	0.018 **	0.008	2.250	0.006	0.039	0.005	0.036
*Direct effects*							
IO→CE	0.227 **	0.067	3.388	0.090	0.354	0.091	0.355
RO→CE	0.203 **	0.067	3.030	0.068	0.333	0.071	0.337
*Total effects*							
IO→CE	0.397 ***	0.058	6.845	0.279	0.507	0.279	0.508
RO→CE	0.370 ***	0.056	6.607	0.261	0.481	0.265	0.483

Note: N = 460, ** *p* < 0.01, *** *p* < 0.001.

**Table 6:** The results of the moderated effects.

Effect Path	Independent Self-Construal			Interdependent Self-Construal		
	Effect	SE	95%CI	Effect	SE	95%CI
IO→SP	0.296 ***	0.055	(0.187, 0.405)	0.834 ***	0.046	(0.743, 0.924)
RO→SP	0.286 ***	0.058	(0.172, 0.399)	0.775 ***	0.048	(0.680, 0.870)
IO→CT	0.246 ***	0.059	(0.130, 0.363)	0.796 ***	0.049	(0.698, 0.893)
RO→CT	0.248 ***	0.058	(0.134, 0.361)	0.826 ***	0.048	(0.731, 0.921)

Note: N = 460, *** *p* < 0.001.

**Table 7:** The results of the moderated chain mediation effects.

Effect Path	Independent Self-Construal			Interdependent Self-Construal		
	Effect	SE	95%CI	Effect	SE	95%CI
IO→SP→CE	0.078	0.025	(0.037, 0.136)	0.221	0.044	(0.138, 0.309)
RO→SP→CE	0.082	0.023	(0.043, 0.131)	0.222	0.046	(0.135, 0.315)
IO→CT→CE	0.052	0.028	(0.001, 0.109)	0.176	0.035	(0.112, 0.249)
RO→CT→CE	0.051	0.024	(0.006, 0.104)	0.183	0.040	(0.110, 0.263)
IO→SP→CT→CE	0.020	0.008	(0.007, 0.039)	0.056	0.019	(0.024, 0.099)
RO→SP→CT→CE	0.019	0.008	(0.007, 0.038)	0.050	0.016	(0.023, 0.087)

Note: N = 460.

**Table 8:** The results of hypothesis testing.

Hypotheses	Result
H1a, H1b	Supported
H2a, H2b	Supported
H3a, H3b	Supported
H4a, H4b	Supported
H5a, H5b	Supported
H6	Supported
H7a, H7b	Supported
H8a, H8b, H8c, H8d	Supported
H9a, H9b	Supported

## Data Availability

The data are available from the corresponding author upon reasonable request.

## Supplementary Materials

The following supporting information can be downloaded at: https://www.mdpi.com/article/10.3390/bs13070541/s1, File S1: Questionnaire on Social Interaction and Customer engagement in social commerce.

## References

[1] Neilpatel. https://neilpatel.com/blog/livestreaming-importance-2018.

[2] Go-Globe. https://www.goglobe.com/live-streaming-statistics.

[3] TechJury. https://techjury.net/blog/livestreaming-statistics/#gref.

[4] Lin Y., Yao D., Chen X. (2021). Happiness Begets Money: Emotion and Engagement in Live Streaming. J. Mark. Res..

[5] Wongkitrungrueng A., Assarut N. (2020). The Role of Live Streaming in Building Consumer Trust and Engagement With Social Commerce Sellers. J. Bus. Res..

[6] Bhattacharyya S., Bose I. (2020). S-commerce:Influence of Facebook Likes on Purchases and Recommendations on a Linked E-commerce Site. Decis. Support. Syst..

[7] Shen J. (2012). Social Comparison, Social Presence, and Enjoyment in the Acceptance of Social Shopping Websites. J. Electron. Commer. Res..

[8] Wang C., Zhang P. (2012). The Evolution of Social Commerce: The People, Management, Technology, and Information Dimensions. Commun. Assoc. Inf. Syst..

[9] Zhou J., Zhou J., Ding Y., Wang H. (2019). The Magic of Danmaku: A Social Interaction Perspective of Gift Sending on Live Streaming Platforms. Electron. Commer. Res. Appl..

[10] Liu H., Xie Q.W., Lou V.W. (2019). Everyday Social Interactions and Intra-individual Variability in Affect:A Systematic Review and Meta-Analysis of Ecological Momentary Assessment Studies. Motiv. Emot..

[11] He A.Z., Liu S.S. (2021). The Influence of Social Media Brand Page Characteristics on Customer Engagement Behavior. J. Northeast. Univ. Soc. Sci..

[12] Li M., Zhang Y.X. (2021). Action Mechanism of E-Servicescape on Customer Engagement: A Non-recursive Model. Financ. Trade Res..

[13] Pongpaew W., Speece M., Tiangsoongnern L. Customer Brand Engagement, Perceived Social Presence, and Brand Trust and Loyalty in Corporate Facebook. Proceedings of the 2016 Annual Conference of the Emerging Markets Conference Board.

[14] Zhang J., Ma Y.R., Jiang S.S. (2021). Can User-Generated Content Quality Affect Customer Brand Engagement?-Research on Virtual Brand Community. Theory Pract. Financ. Econ..

[15] Fan G.G., Wu M. (2019). Research on the Influence of Virtual Brand Perceived Community Support on Customer Engagement Behavior. Soft Sci..

[16] Xue J., Liang X., Xie T., Wang H. (2020). See now, act now: How to interact with customers to enhance social commerce engagement?. Inf. Manag..

[17] Samala N., Katkam B.S. (2019). Fashion Brands are Engaging the Millennials: A Moderated Mediation Model of Customer Brand Engagement, Participation, and Involvement. Young Consum..

[18] Samarah T., Bayram P., Aljuhmani H.Y., Elrehail H. (2021). The Role of Brand Interactivity and Involvement in Driving Social Media Consumer Brand Engagement and Brand loyalty: The Mediating Effect of Brand Trust. J. Res. Interact. Mark..

[19] Fei M., Tan H., Peng X., Wang Q., Wang L. (2021). Promoting or Attenuating? An Eye-tracking Study on the Role of Social Cues in E-commerce Livestreaming. Decis. Support. Syst..

[20] Guo L., Hu X., Lu J., Ma L. (2021). Effects of Customer Trust on Engagement in Live Streaming Commerce: Mediating Role of Swift Guanxi. Internet Res..

[21] Becker G.S. (1974). A Theory of Social Interactions. J. Political Econ..

[22] Chen Y.X., Gao X.T., Wen Y.Y. (2021). Research on Mutual Trust between Buyers and Sellers in Online Live Shopping Mode. Chin. J. Manag. Sci..

[23] Lee K., Nass C. (2005). Social-psychological Origins of Feelings of Presence: Creating Social Presence with Machine-generated Voices. Media Psychol..

[24] Zhao H.X., Wang X.H., Zhou B.G. (2015). Relationship among Interaction, Presence and Consumer Trust in B2C Online Shopping. Manag. Rev..

[25] Gunawardena C.N. (1995). Social Presence Theory and Implications for Interaction and Collaborative Learning in Computer Conferences. Int. J. Educ. Telecommun..

[26] Keng C.J., Chang W.H., Chen C.H., Chang Y.Y. (2016). Mere Virtual Presence with Product Experience Affects Brand Attitude and Purchase Intention. Social. Behav. Personal. An. Int. J..

[27] Zhou Y.S., Tang S.H., Xiao J. (2021). Research on Consumers’ Purchase Intention on E-commerce Livestreaming Platforms—Based on the Perspective of Social Presence. Contemp. Econ. Manag..

[28] Li X., Yang C., Wang S. (2023). Research on the Impact of Intercustomer Social Support on Customer Engagement Behaviors in Virtual Brand Communities. Behav. Sci..

[29] Mehrabian A., Russell J. (1974). An Approach to Environmental Psychology.

[30] Sherman E., Mathur A., Smith R.B. (1997). Store Environment and Consumer Purchase behavior: Mediating Role of Consumer Emotions. Psychol. Mark..

[31] Van Doorn J., Lemon K.N., Mittal V., Nass S., Pick D., Pirner P., Verhoef P.C. (2010). Customer Engagement Behavior: Theoretical Foundations and Research Directions. J. Serv. Res..

[32] Hollebeek L.D., Glynn M.S., Brodie R.J. (2014). Consumer Brand Engagement in Social Media: Conceptualization, Scale Development and Validation. J. Interact. Mark..

[33] Gao X.Y., Li Q., Xu X.Y., Lv S. (2021). The Influence of Co-Viewers on Viewers’ Purchase Intention in Live Streaming Commerce. J. Xi’an Jiaotong Univ. Soc. Sci..

[34] Kim S., Park H. (2013). Effects of Various Characteristics of Social Commerce (s-commerce) on Consumers’ Trust and Trust performance. Int. J. Inf. Manag..

[35] Yahia I.B., Alneama N., Kerbache L. (2018). Investigating the Drivers for Social Commerce in Social Media Platforms: Importance of Trust, Social Support and the Platform Perceived Usage. J. Retail. Consum. Serv..

[36] Gibreel O., Alotaibi D.A., Altmann J. (2018). Social Commerce Development in Emerging markets. Electron. Commer. Res. Appl..

[37] Zhao B.G., Wang Y.F. (2021). The Influence of E-commerce Anchor Characteristics on Consumer Purchase Intention. Commer. Res..

[38] Wang J.B., Man S.S., Dun S. (2022). The Relationship Between Trust Transfer and Intention of Continuous Use in Sharing Economy: The Moderating Role of Transboundary Network Effect. Commer. Res..

[39] Shin D.H., Shin Y.J. (2011). Consumers’ Trust in Virtual Mall Shopping: The Role Of Social Presence and Perceived Security. Int. J. Hum. Comput. Interact..

[40] Singelis T.M. (1994). The Measurement of Independent and Interdependent Self-Construals. Pers. Soc. Psychol. Bull..

[41] Wang W.C., Zhao Y.F., Xiao Z.L., Chen B. (2022). The Influence of Self-Construal on Group Reference Effects. J. Psychol. Sci..

[42] Duan K., Wang D.H., Yao T., Qiu Q. (2018). The Influence of Self-construction on the Consumers’ Promotions Preference. J. Manag. Sci..

[43] Yu Z.P., Zeng H., Hao L.G. (2022). An Empirical Research on the Impact of Participation Atmosphere on Online Participation Intention: From the Perspective of Social Exchange Theory. J. Ind. Eng. Eng. Manag..

[44] Tu K., Yang X.C., Su X., Ou X.C. (2020). The Influence of Supplier User’s Role Stress on Continuous Value Co-creation Behavior in the Sharing Economy: A Mediated Moderation Model. Nankai Bus. Rev..

[45] Bagozzi R.P., Yi Y. (1988). On the Evaluation of Structural Equation Models. J. Acad. Mark. Sci..

[46] Im J., Qu H.L. (2017). Drivers and Resources of Customer Co-Creation: A Scenario-Based Case in the Restaurant Industry. Int. J. Hosp. Manag..

[47] Hair J.F., Black W.C., Babin B.J., Anderson R.E. (2010). Multivariate Data Analysis.

[48] Wu S.H., Huang S.C.T., Tsai C.Y.D., Lin P.Y. (2017). Customer Citizenship Behavior on Social Networking Sites the Role of Relationship Quality, Identification, and Service Attributes. Internet Res..

[49] Preacher K.J., Hayes A.F. (2008). Asymptotic and Resampling Strategies for Assessing and Comparing Indirect Effects in Multiple Mediator Models. Behav. Res. Methods..

[50] Cohen P., West S.G., Aiken L.S. (2014). Applied Multiple Regression/Correlation Analysis for the Behavioral Sciences.

[51] Meng J., Wang Y., Wang X., Hao Z. (2023). How Corporate Social Responsibility Influences Business Model Innovation. J. Liaoning Univ..

[52] Kang K., Lu J., Guo L., Li W. (2021). The Dynamic Effect of Interactivity on Customer Engagement Behavior through Tie Strength: Evidence from Live Streaming Commerce Platforms. Int. J. Inf. Manag..

