# Supplementary material for: Impact of Social Interaction on Customer Engagement in China’s Social Commerce—A Moderated Chain Mediation Model
Source: Behav Sci (Basel). 2023 Jun 28;13(7):541. doi: 10.3390/bs13070541 (PMC10376239; doi:10.3390/bs13070541)
Supplement: Supplementary file 1 [file behavsci-13-00541-s001.zip › behavsci-2409770-supplementary.pdf]

Supplementary Materials

Questionnaire on Social Interaction and Customer engagement in social commerce

Dear Respondent,

Thank you for taking the time to complete this questionnaire. This academic survey examines social interaction and customer engagement in social commerce. Your responses are anonymous and will be kept strictly confidential. The data will be used solely for scholarly research purposes. There are no "right" or "wrong" answers; please respond based on your honest thoughts and opinions. Your candid feedback is greatly appreciated.

Part I:

Please read the instructions before answering the questions.

Description of Social Commerce
Social commerce relies primarily on interpersonal relationship networks. Using social networks, online media, and other communication channels, social commerce facilitates the buying and selling of goods through social interaction, user-generated content, and other means. The social commerce can be categorized into three categories. The first category integrates social features into traditional e-commerce platforms, such as Taobao's "Weitao" and Jingdong's "Discovery" - online communities that enable users to share shopping experiences. The second category integrates shopping features into social media platforms. This includes the array of mini shopping apps and merchant service accounts in WeChat that offer direct purchasing, as well as the shopping functions found on platforms like Weibo, Xiaohongshu, and TikTok. The third category involves cross-platform social shopping, where social media and e-commerce platforms are closely integrated. That is, users use their social connections on social media to complete purchases on e-commerce platforms. They share shopping information with friends, play interactive shopping games together, and more—such as by joining group buys and giving gifts through Pinduoduo's friend circles.

1. Do you have experience participating in social commerce platforms (including registration/browsing/posting/commenting/participating in interactions/shopping).:

□Yes

□No

2. Please specify the category of social commerce platforms you are most familiar with.

□ Category One: integrating social features into traditional e-commerce platforms

□Category Two: integrating shopping features into social media platforms

□ Category Three: cross-platform social shopping, where social media and e-commerce platforms are closely integrated.

□ Others

Part II: Rating Scales

Choose from the following statements based on the specific category of social commerce platforms with which you are most familiar. (1 = Strongly Disagree, 2 = Disagree, 3 = A Little Disagree, 4 = Neutral, 5 = A Little Agree, 6 = Agree, 7 = Strongly Agree)

(1) Indicate the extent to which you agree/disagree with the following statements.

		Strongly Disagree			Neutral			Strongly Agree
1	Social commerce platforms make it effortless for me to access useful product information.	1	2	3	4	5	6	7
2	Social commerce platforms allow me to gain deeper insight into a product's attributes.	1	2	3	4	5	6	7
3	Social commerce platforms empower me to share valuable product insights with my communities.	1	2	3	4	5	6	7
4	I will seek help from others within my social commerce platform circles for product-related questions.	1	2	3	4	5	6	7
5	I will post messages on social commerce platforms and get responses from others.	1	2	3	4	5	6	7
6	I frequently connect through open dialogue with others on the social commerce platforms, bonding over shared interests and building relationships.	1	2	3	4	5	6	7
7	I frequently participate in discussions and events within my social commerce platforms, coming together with others to collaborate, support each other, and shape meaningful outcomes.	1	2	3	4	5	6	7

(2) Indicate the extent to which you agree/disagree with the following statements.

		Strongly Disagree			Neutral			Strongly Agree
8	Social commerce gives me a sense of being immersed and present.	1	2	3	4	5	6	7
9	Social commerce gives virtual goods a feeling of authenticity for me	1	2	3	4	5	6	7
10	Social commerce gives virtual marketplaces a feeling of authenticity for me.	1	2	3	4	5	6	7

(3) Indicate the extent to which you agree/disagree with the following statements.

		Strongly Disagree			Neutral			Strongly Agree
11	I feel that the offerings from merchants on social commerce platforms have a guaranteed quality.	1	2	3	4	5	6	7
12	I have a high degree of trust in the ability of merchants on social commerce platforms to integrate resources.	1	2	3	4	5	6	7
13	I have confidence in the credibility of merchants on social commerce platforms.	1	2	3	4	5	6	7

(4) Indicate the extent to which you agree/disagree with the following statements.

		Strongly Disagree		Neutral			Strongly Agree	
14	When I need to shop, social commerce platforms come to mind for me.	1	2	3	4	5	6	7
15	The shopping scenario and atmosphere on social commerce platforms is interesting.	1	2	3	4	5	6	7
16	I have a deep impression of interacting with merchants and audiences on social commerce platforms.	1	2	3	4	5	6	7

(5) Indicate the extent to which you agree/disagree with the following statements.

		Strongly Disagree		Neutral			Strongly Agree	
17	I am easily influenced by other people's ideas.	1	2	3	4	5	6	7
18	Independent personality traits are not very important to me	1	2	3	4	5	6	7
19	I am quite willing to remain consistent with those around me in many ways.	1	2	3	4	5	6	7

Part III: Basic Information

1. Gender

□ Men □ Women

2. Age

□ Under 18 □ 18–25 □ 26–35 □ 36–45□ Over 45

3. Educational level

□ High school/technical secondary school and below
□ College degree
□ Bachelor's degree
□ Master's degree and above

4. Years of watching live streaming

□ Less than half a year □ Half a year to two years □ Two years and above

5. Platforms online duration

□ less than 1 hour per day □ 1-2 hours per day □ 2-3 hours per day □ More than 3 hours per day

Thank you for completing this survey.
